# Optoelectronic Angular Displacement Measurement Technology for 2-Dimensional Mirror Galvanometer

**DOI:** 10.3390/s22030872

**Published:** 2022-01-24

**Authors:** Shao-Kang Hung, Yu-Hsin Chung, Cheng-Lung Chen, Kai-Hung Chang

**Affiliations:** Department of Mechanical Engineering, National Yang Ming Chiao Tung University, No. 1001, University Road, Hsinchu 30010, Taiwan; s0851013.me08g@nctu.edu.tw (Y.-H.C.); bruce.me08g@nctu.edu.tw (C.-L.C.); mxc0451002.me04g@nctu.edu.tw (K.-H.C.)

**Keywords:** angle sensor, optoelectronic sensor, parametric design

## Abstract

The mirror galvanometer is a crucial component of laser cutters/engravers. Novel two-dimensional mirror galvanometers demonstrate less trajectory distortion than traditional one-dimensional ones. This article proposes an optoelectronic sensor that measures a mirror’s inclinations in two dimensions simultaneously. The measuring range, resolution, and sampling rate are ±10°, 0.0265°, and 2 kHz, respectively. With the proposed sensor, a closed-loop control can be further implemented to achieve precision laser machining. Its compact size and low cost meet the requirements of miniature laser engravers, which have become popular in recent years.

## 1. Introduction

In 2017, the first miniature laser engraver (MLE) [1] was invented, and a new consumer product called the “personal laser tool” was born. Compared with industrial laser engravers, MLEs do not have high specifications, but their portability (153 g/5^3^ cm^3^) and affordability are attractive. Analyzing the internal structure of a typical MLE, Cubiio, we found that the key to its tiny size and low cost is replacing the bulky f-theta lens with a virtual lens [2]. This patented technique requires a ±20° laser scanning range, i.e., a ± 10° mirror tilting range, and then projects to a 100 mm-square workspace. The un-interpolated image contains 500 × 500 pixels; therefore, the objective resolution is one five-hundredth of the full range. 

**Figure 1:**
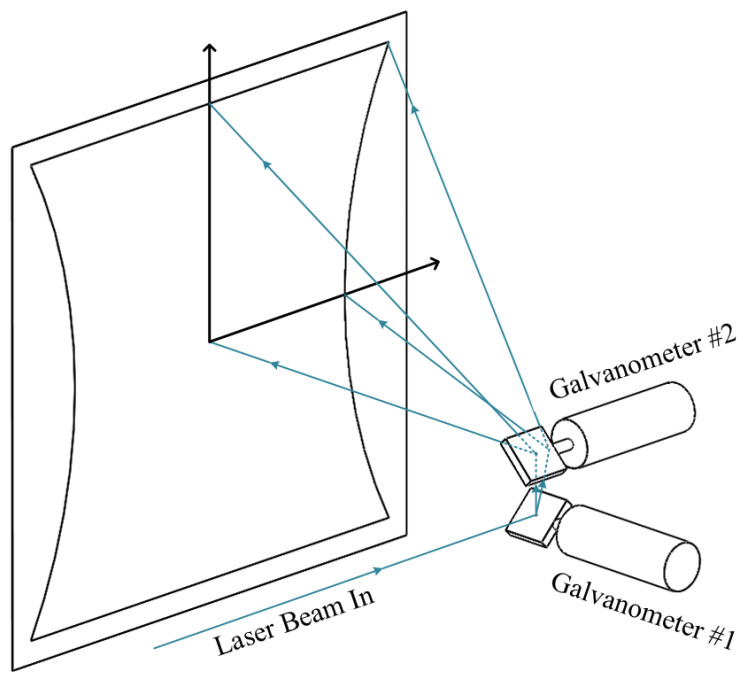
The optical arrangement inside conventional laser engravers.

Figure 1 illustrates the arrangement of optical elements inside conventional laser engravers. Two 1D mirror galvanometers are installed orthogonally. The laser beam is reflected first by the horizontal scanner and then by the vertical scanner. Multiple reflections attenuate the laser power, and the different lengths between horizontal and vertical optical paths cause a pillow-shaped distortion [3,4], as shown in Figure 1. To avoid the aforementioned disadvantages, 2D mirror galvanometers were developed. In these, a single mirror that can tilt two-dimensionally reflects the laser beam only once.

Atomic force microscopes [5,6] and CD/DVD pickup heads [7,8] utilize quadrant photodetectors (QPD) [9,10] to achieve 2D angular-displacement sensing with ultra-high resolution, but their measuring ranges are limited. Micro-opto-electromechanical systems (MOEMS) [11] benefit from the development of semiconductor integrated circuits and microelectromechanical systems (MEMS) [12,13]. MOEMS scanning mirrors have enjoyed great commercial success in the field of display. The most famous example is that of Texas Instruments’ digital micromirror devices [14], which are widely used in projectors. Combining QPD and MOEMS, 2D scanning mirrors with embedded sensors have been built, modeled, and analyzed [15,16,17]. Their specifications, listed in Table 1, show that MOEMS scanning mirrors are compact with good resolution, but their sensing ranges cannot meet the requirements of MLE. Furthermore, their small mirrors cannot withstand the heat produced by the high-power laser. Another method for measuring 2D tilt is by utilizing optical fibers [18,19,20,21], but they are usually bulky due to the limitations of the bending radius. Therefore, MOEMS technology is not suitable for use in MLE applications. To increase the sensing range, the scale of the sensor needs to be larger. Printed circuit board (PCB) technology with surface-mount devices (SMD) meets this requirement and has lower developing costs than MOEMS technology. With the abovementioned advantages, PCB-based sensing technology has also been applied to encoders [22] and robot joints [23,24,25].

In this article, a PCB-based sensor that satisfies the needs of MLEs has been designed and proposed. After introducing the details of the proposed sensor in Section 2, an algorithm that converts four signals into two angles is developed in Section 3. The experimental validations and the overall performance are summarized, and conclusions are drawn in the final sections.

## 2. Sensor Design and Operation Principle 

**Figure 2:**
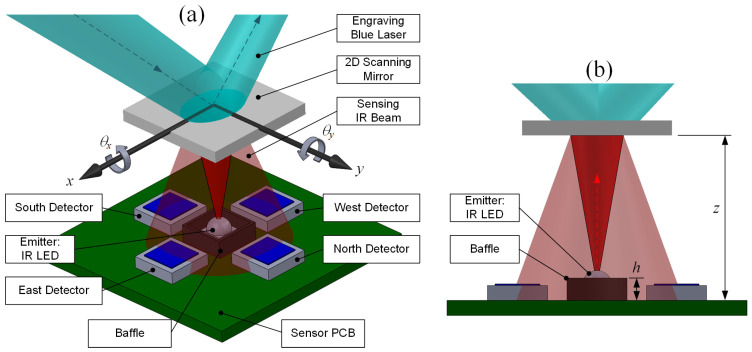
(**a**) The structure of the proposed sensor. The 2D scanning mirror manipulates the high-power blue laser. On the back, four detectors receive different infrared intensities according to the mirror’s orientation $\left(\theta_{x},\theta_{y}\right)$. (**b**) Side-view of the proposed sensor. *h* and *z* represent the height of the baffle and the mirror–PCB distance, respectively.

This sensor is designed to transduce the angles of inclination, *θ_x_* and *θ_y_*, of a 2D scanning mirror into four electronic signals, which will be further acquired and processed into two digital values to represent *θ_x_* and *θ_y_*. The physical mechanism of operation is illustrated in Figure 2 and is described in detail in the following section.

### 2.1. Element Selection

In the test setup, a double-sided coated mirror (RB4550, Rocoes, Taiwan) with 24-layer thin films is used to reflect a 1.6 W high-power blue laser (TB450B, Osram, Munich, Germany) to engrave the target piece. The mirror’s reflectivity is greater than 98% from a 450 to 500 nm wavelength at its designed angle of incidence, 45°. High reflectivity implies low absorptivity, which keeps the mirror below its safe temperature of 150 °C. The details of the 2D actuator that manipulates the mirror are beyond the scope of this article and will be reported in other literature. A center-located LED emits a 940 nm infrared ray to the back side of the mirror. Our selected LED (VSMB14940, Vishay, Malvern, PA, USA) has the narrowest “angle of half-intensity” in the market of 9° for the purposes of high sensitivity. Four surrounded phototransistors receive the unbalanced infrared ray reflected by the tilting mirror. Our selected phototransistors (SFH3400, Osram, Munich, Germany) have a linear response to the incident angle. To ensure producibility and low cost, off-the-shelf SMD components are preferred. In addition, all tiny elements are placed as close together as possible because a miniature sensor PCB is anticipated.

**Figure 3:**
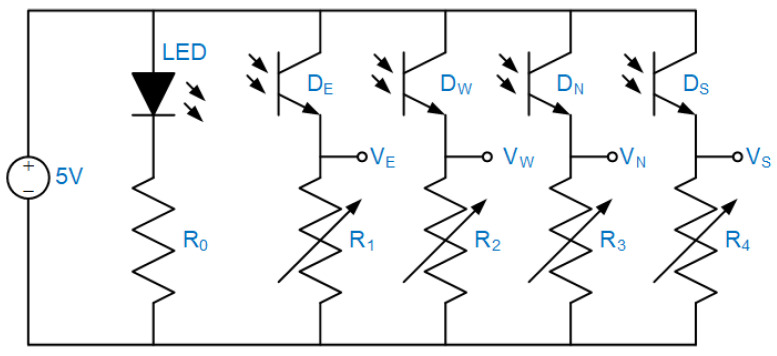
The schematic drawing of the sensor circuit, powered by 5 V of DC.

**Figure 4:**
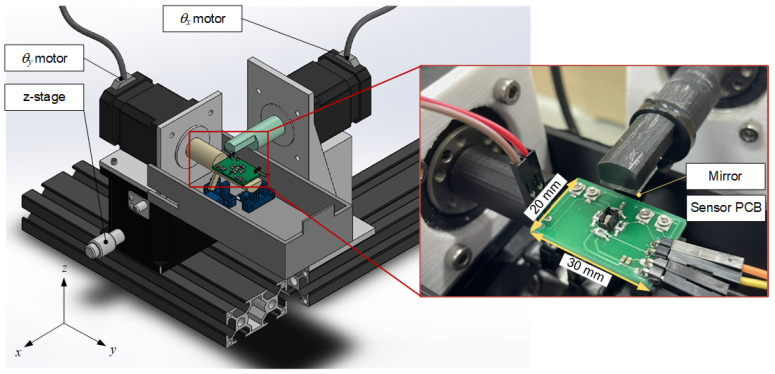
Test-bench consisting of two motor-driven shafts and a translational z-stage.

Figure 3 shows the schematic drawing of the sensor circuit. The resistor R_0_ keeps the LED working at its nominal voltage of 1.24 V and current of 20 mA. The variable resistors R_1–4_ are tuned to modulate the output signals, $V_{E},\;V_{W},\;V_{N},V_{S},$ of four detectors because they may have individual characteristics. All elements are soldered onto a PCB, as shown in Figure 4. Three design parameters, the height of the baffle (h), phototransistor-connected resistance (R_1–4_), and the vertical distance between the central emitter and the mirror (z), will be quantified by practical tests in the following section.

### 2.2. Test Bench

A test bench was built to achieve the aforementioned design parameters. To generate a relative 2D tilting motion between the mirror and the sensor board, two 5-phase stepping motors (PK543BW-H50S, Oriental Motor, Tokyo, Japan) with harmonic gears were installed orthogonally, as illustrated in Figure 4. Motors X and Y rotate the sensor around the *x*-axis and the mirror around the *y*-axis, respectively. The precision displacement stage is equipped to manually adjust the vertical distance between the central emitter and the mirror, *z*. Combining the stepping motor’s resolution, 500 pulse/rev, and the backlash-less harmonic gear [26] with a 50:1 reduction ratio, ultrahigh angular accuracy at 0.0144° can be achieved. The stepping motors are driven by compatible drivers (CRD507-K, Oriental Motor, Tokyo, Japan) and are controlled by a data acquisition card (USB-6341, National Instruments, Austin, TX, USA), which also collects the analog voltage signals from four phototransistors. The whole system is hosted by a computer (2.5 GHz, Intel i5 CPU) and programmed by a graphic language (LabVIEW, National Instruments, Austin, TX, USA). Finally, this apparatus is hooded by an opaque box to shield off the ambient light.

### 2.3. Height of the Baffle

**Figure 5:**
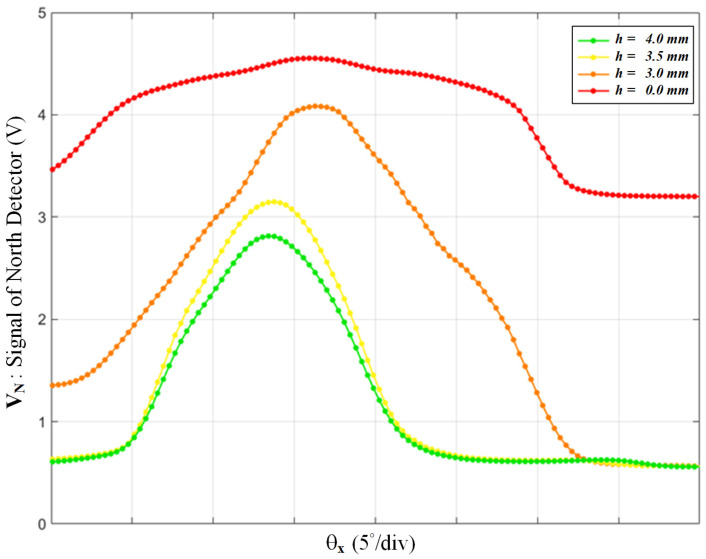
The north detector’s signal over *θ_x_*, with the different height values of the baffle, which blocks the sideward leakage from the emitter LED.

Although the infrared LED emits a narrow beam upward, there is still sideward leakage that affects the surrounded phototransistors directly. Therefore, a baffle is needed to block the sideward leakage. The 3D-printed baffle is made of light-hardening resin with a thin rectangular wall just a little bigger than the emitter’s footprint. Its color is matte black to absorb the scattered light. Many baffles with different height values were printed for the following test, under the default conditions of R_1–4_ = 200 Ω and z = 10 mm. Using the test-bench, we scanned *θ_x_* and plotted the signal V_N_ in Figure 5. In the unblocked case, i.e., *h* = 0, the signal was always above 3 V due to the sideward leakage. The height of the emitter LED was 2 mm; therefore, the baffle’s height started from 3 mm. In the case where *h* = 3 mm, the residual sideward leakage caused the signal to be a little distorted. In the case where *h* = 4 mm, the baffle was too high and suppressed the dynamic range of the signal. Therefore, *h* is decided at 3.5 mm, which is also suitable for the other three signals.

### 2.4. Phototransistor-Connected Resistor

**Figure 6:**
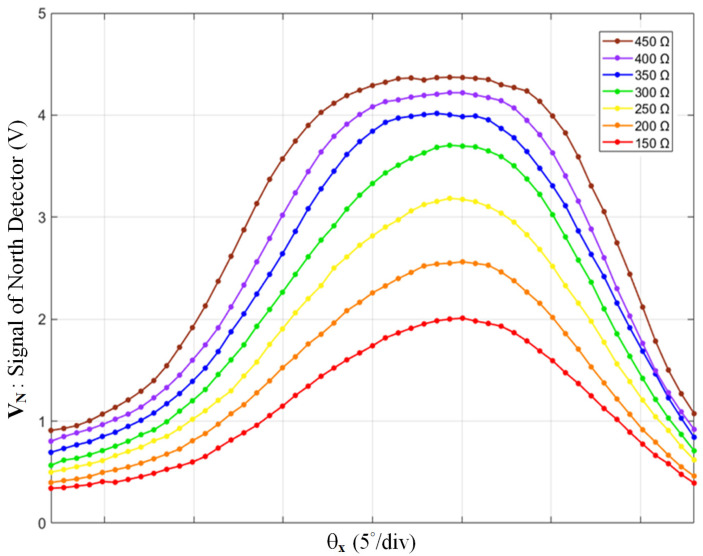
The north detector’s signal over *θ_x_*, with different values of phototransistor-connected resistance.

The default resistance, 200 Ω, was estimated according to the datasheets of the emitter and the phototransistor. This resistance can be refined under conditions where *h* = 3.5 mm, the most suitable height of the baffle. Using the test-bench, we scanned at *θ_x_* and have plotted the signal V_N_ in Figure 6. By tuning the variable resistor R_1_ from 150 to 450 Ω, the response curves are very different. Low resistance causes a low slope and poor sensitivity; on the other hand, when set too high, the resistance causes saturation, i.e., a flat region. This test established that the most suitable R_1_ ranged from 250 to 300 Ω. By repeating the procedure for the other three signals, suitable ranges for R_2–4_ can be obtained as well.

### 2.5. Emitter-Mirror Distance

**Figure 7:**
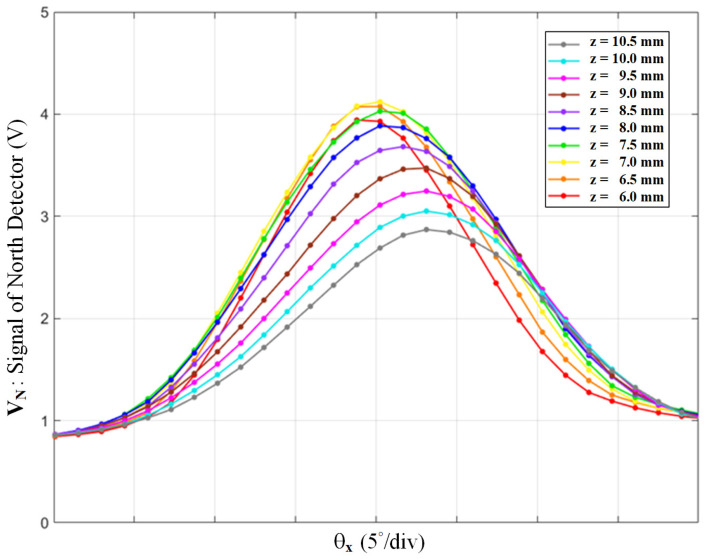
The north detector’s signal over *θ_x_*, with different values of the PCB-mirror distance.

The final parameter, PCB-mirror distance, z, can be decided according to the conditions refined earlier, h = 3.5 mm and R_1_ = 300 Ω. By fine-tuning at the precision displacement stage, we scanned z from 6 to 10.5 mm and plotted the signal V_N_ shown in Figure 7 in the same manner. As the PCB-mirror distance goes further, the peak value of the signal goes up and then down. From z = 6.5 to 7.0 mm, the curves show a maximal dynamic range with good linearity. In summary, the optimal design parameters can be seen in Table 2. With suitable design parameters, the proposed sensor can generate four 2D bell-shaped signals over *θ_x_* and *θ_y_*. An algorithm that converts the four signals into *θ_x_* and *θ_y_* will be developed in the next section.

## 3. Inverse Mapping Algorithm

A typical QPD method inversely maps four signals to two angular displacements using Equation (1):

(1) $$(\theta_{x},\;\theta_{y})=\left(\frac{C_{x}(V_{N}-V_{S})}{V_{E}+V_{W}+V_{N}+V_{S}},\frac{C_{y}(V_{E}-V_{W})}{V_{E}+V_{W}+V_{N}+V_{S}}\right)$$

where $V_{E},\;V_{W},\;V_{N},V_{S}$ are the signals measured by detectors in the east, west, north, and south; $C_{x}$ and $C_{y}$ are the coefficients obtained by calibration. However, these equations are only valid when the angular displacements are small. If the mirror’s scanning range is as large as ± 10°, the behavior is no longer independent and linear. A more complicated inverse mapping algorithm has been developed below.

**Figure 8:**
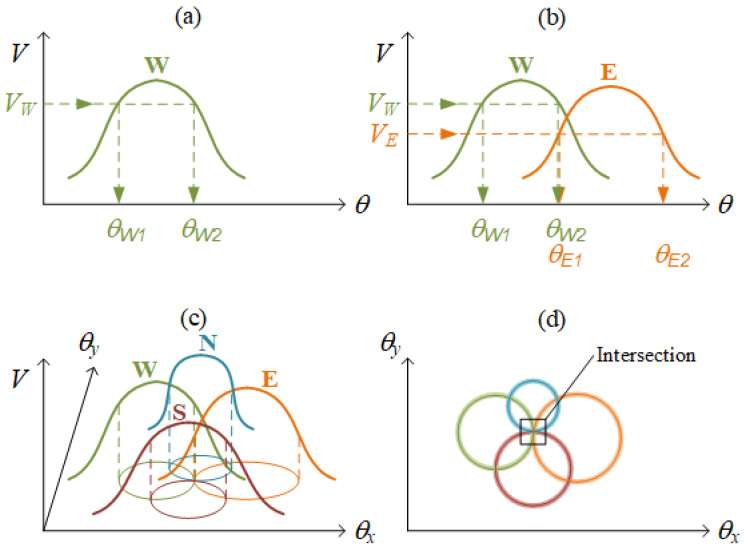
(**a**) One detector for one dimension. (**b**) Two detectors for one dimension. (**c**) QPD: four detectors for two dimensions. (**d**) The intersection of four hoops contains multiple candidates.

When approaching a simplified 1D case in Figure 8a, only one bell-shaped signal has been pre-collected in the database. During sensing, a measured voltage, $V_{W}$, projects to two possible candidates, $\theta_{W1}$ and $\theta_{W2}$, but we do not know which one is true. To obtain a unique θ output, more information is necessary. If there is one more detector with a bell-shaped signal like Figure 8b, $V_{E}$ projects to $\theta_{E1}$ and $\theta_{E2}$. The intersection of $\left\{\theta_{W1},\;\theta_{W2}\right\}$ and $\left\{\theta_{E1},\;\theta_{E2}\right\}$ yields the unique output, $\theta_{W2}=\theta_{E1}$. 

In the same manner, for a 2D case, four detectors with four bell-shaped signals are pre-collected as in Figure 8c. When the mirror turns to (*θ_x_*, *θ_y_*) during sensing, four measured voltages project to four circles on the solution domain. The correct sensing result, (*θ_x_*, *θ_y_*), is located within the intersection of these four circles. In a realistic measurement with noise, however, a level of tolerance must be allowed when screening candidates because two measurements are rarely exactly equal to each other. Therefore, as illustrated in Figure 8d, four circles become four hoops, the intersection of which contains multiple candidates. A further averaging procedure will be applied to produce a single result.

**Figure 9:**
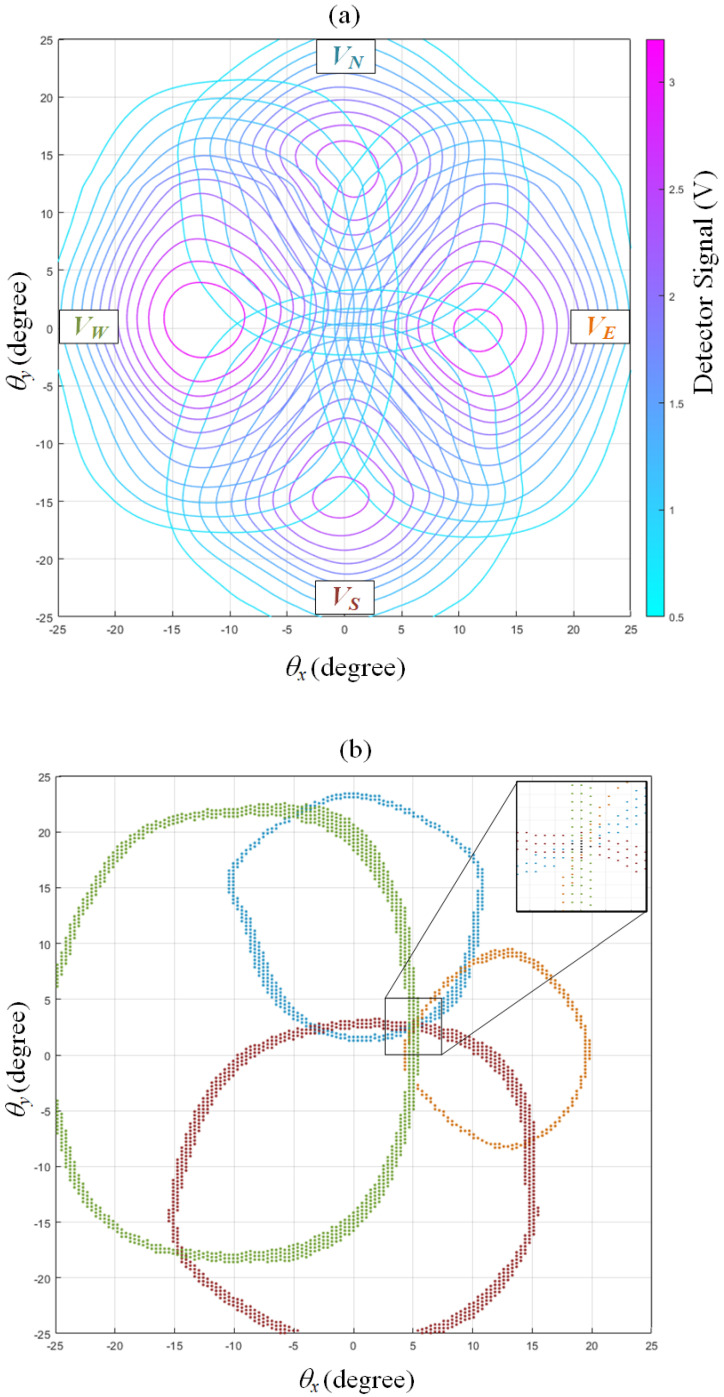
(**a**) Four bell-shaped signals pre-collected by our proposed sensor. (**b**) For a realistic measurement with noise, $V_{E},\;V_{W},\;V_{N},V_{S}$ are inversely mapped to four hoops. Orange, green, blue, and red dots represent the candidates, projected from $V_{E},\;V_{W},\;V_{N},V_{S}$, respectively. Black dots represent their intersection, as shown in the zoomed region.

Figure 9a illustrates the distribution of four bell-shaped signals, pre-collected by our proposed sensor. As explained before, in a realistic measurement with noise, there are multiple candidates within the intersection, as shown in Figure 9b. To obtain meaningful $\left(\theta_{x},\theta_{y}\right)$ among these candidates, a weighted average is applied, as in Equation (2):

(2) $$(\theta_{x},\theta_{y})=\frac{\sum_{i=1}^{n}[w_{i}(\theta_{xi},\theta_{yi})]}{\sum_{i=1}^{n}w_{i}}$$

where *i* and *n* are the index and the number of candidates; $\left(\theta_{xi},\theta_{yi}\right)$ is the location of the *i*th candidate; and $w_{i}$ is the weight defined by Equation (3):

(3) $$w_{i}={\left|V_{E}-V_{Ei}\right|}^{-1}+{\left|V_{W}-V_{Wi}\right|}^{-1}+{\left|V_{N}-V_{Ni}\right|}^{-1}+{\left|V_{S}-V_{Si}\right|}^{-1}$$

where $V_{E},\;V_{W},\;V_{N},V_{S}$ are the voltages presently measured by detectors in the east, west, north, and south, respectively; $V_{Ei},\;V_{Wi},\;V_{Ni},V_{Si}$ are the *i*th candidate’s voltages as pre-collected in the database. The four voltage values, grouped as a set, can be perceived as the “fingerprint” of a candidate. If a candidate’s fingerprint is more similar to the present measurement, its weight should be stronger. Conversely, the weight should be weaker if the fingerprint is less similar to the present measurement. To implement the above idea mathematically in Equation (3), the weight is designed as the reciprocal summation of the absolute value between a candidate’s recorded voltages and presently measured voltages.

There are other mathematical approaches to designing the weighting, e.g., Equation (4), which generates similar fusion results; however, the squaring operations take some time and seriously slow down the overall speed. To achieve a prompt response, the weighting is designed as in Equation (3), with simple and quick mathematical operations. The following experiments show that Equations (2) and (3) can work effectively and efficiently:

(4) $$w_{i}={\left(V_{E}-V_{Ei}\right)}^{-2}+{\left(V_{W}-V_{Wi}\right)}^{-2}+{\left(V_{N}-V_{Ni}\right)}^{-2}+{\left(V_{S}-V_{Si}\right)}^{-2}$$

## 4. Experiment

**Figure 10:**
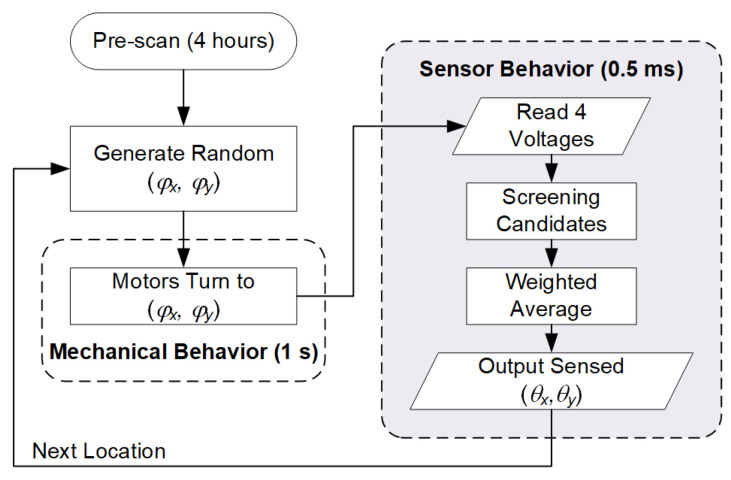
The flow chart for testing the proposed sensor.

To verify the performance of the proposed sensor, experiments were executed according to the flow chart Figure 10. The thermal equilibrium, i.e., steady voltage readings, can be achieved after 1 min of warmup since the total heat dissipation is lower than 0.5 W.

STEP 1: Pre-scan. Every fabricated sensor board needed to be 2D-scanned by our test bench, and an associated 4-peak topography was pre-collected in its database. In the record, every $\left(\theta_{x},\theta_{y}\right)$ yields four featured voltage values as a unique fingerprint. 

STEP 2: Within ±15°, we generated a random location $\left(\varphi_{x},\varphi_{y}\right)$ to be tested.

STEP 3: We commanded the motors of the test bench to turn to $\left(\varphi_{x},\varphi_{y}\right)$.

STEP 4: Screening candidates. During sensing, four voltages were inputted and then mapped to four hoops, as illustrated in Figure 8. Several candidates were quickly selected by intersecting these four hoops.

STEP 5. Weighted average. Equations (2) and (3) were calculated to produce $\left(\theta_{x},\theta_{y}\right)$ as the sensor output, then to loop back to STEP 2 for the next location to be tested. The difference between the actual location $\left(\varphi_{x},\varphi_{y}\right)$ and the sensor output $\left(\theta_{x},\theta_{y}\right)$ is defined as the sensing error (Equation (5)):

(5) $$(err_{x},\;err_{y})=\left(|\varphi_{x}-\theta_{x}\left|,\;|\varphi_{y}-\theta_{y}\right|\;\right)$$

**Figure 11:**
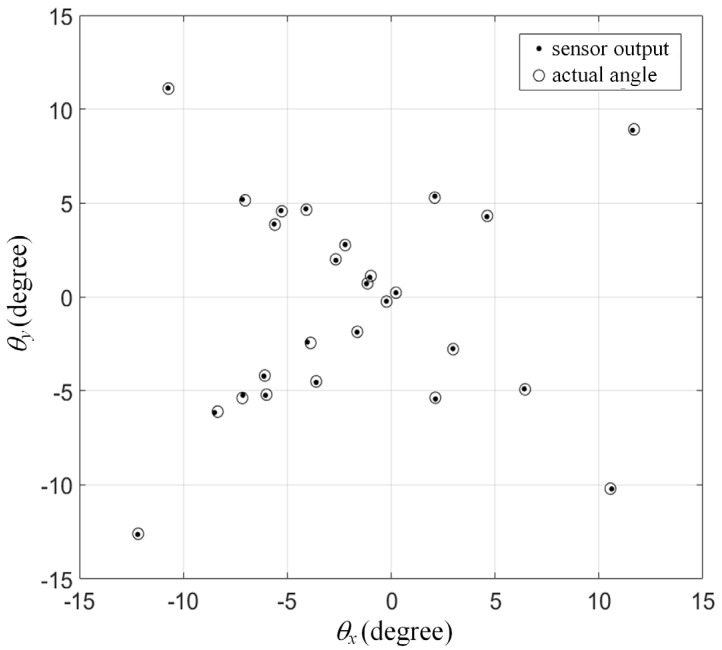
Experimental result: actual angles versus sensor outputs.

STEP 1 takes 4 h to complete because of the high-density data. This time-consuming procedure can be treated as a form of calibration. STEP 3 is a mechanical behavior that takes about 1 s. STEPS 4 and 5 represent a sensor behavior that takes 0.5 ms, i.e., at a 2000 Hz sampling rate. The experimental results are plotted in Figure 11. The sensor outputs are close to the actual locations within a ± 15° test range. We tested a ± 20° range and found that the error increased seriously when the locations were outside ± 15°. To be on the safe side, the range of the proposed sensor is limited to ± 10°. Within the range, the average of errors is 0.0265° in both dimensions. Similar to the idea of a “signal-to-noise ratio”, the resolution of the proposed sensor is defined as “range-to-error ratio”, which is equal to 755. The details of the performance of the proposed sensor are listed in Table 3, which meets the goals of MLE, as listed in Table 1.

## 5. Discussion 

The main novelty of this research is to transfer QPD from a MEMS to a PCB platform. Thus, this research achieves three advantages: a wider sensing range, higher laser power, and a shorter development period.

This research has demonstrated the widest possible sensing range, ± 10°, among the surveyed studies in Table 1. With a doubled scanning range, an MLE can cover a workspace with a half-projection distance, which implies a smaller focusing spot and higher energy density. A small laser spot, cooperated with 0.0265° accuracy, achieves good engraving quality. On the other hand, a high energy density plus a 2000 Hz sampling rate leads to a fast engraving speed. It is definite that the above performance cannot compete with expensive industrial laser engravers, but the proposed sensor can be used in cost-effective MLEs. The μm-level mirrors in MEMS cannot withstand the heat produced by a powerful laser. Thus, MEMS galvanometers are only suited to laser-displaying applications. Our PCB-based system can cooperate with centimeter-level mirrors, which suit more powerful laser engravers. In terms of the development period, a PCB-based layout/component can be modified in a month. In contrast, a MEMS system involving several photomasks would take far longer to be redesigned.

## 6. Conclusions and Outlook 

Our proposed angular displacement sensor can measure the tilt motion of a scanning mirror around two axes simultaneously. This PCB-based sensor consists of off-the-shelf, tiny SMD components, and demonstrates the advantages of a large sensing range, compact size, and cost-effectiveness. The experiments show that the sensing range, averaged error, range-to-error ratio, and sampling rate are ± 10°, 0.0265°, 755, and 2000 Hz, respectively. With the above advantages and performance, the proposed sensor can be utilized in the next generation of miniature laser engravers. In the future, the pulse emission technique with lock-in amplifiers can be adopted to enhance the signal-to-noise ratio for better resolution, which makes it possible for this sensor to be further utilized in industrial laser cutters/engraves, but the cost would necessarily be higher. The tradeoff between accuracy and cost is always inevitable.

## Figures and Tables

**Table 1:** Scanning mirrors with embedded sensors made by QPD and MOEMS, as reported in the surveyed literature.

	Sawada et al. [15]	Cheng et al. [16]	Liu et al. [17]	Goals of MLE [1]
Dimension(s)	1D tile 1D linear	2D tilt	2D tilt	2D tilt
Sensing range	±2.5°	±5°	±5°	≥±10°
Resolution	NA	0.238°/mV	0.0067°	≤0.04°
Mirror size	~1 × 1 mm^2^	2 × 2 mm^2^	1.4 × 1 mm^2^	≥8 × 5 mm^2^
Device footprint	1.5 × 1.5 mm^2^	3 × 3 mm^2^	11.4 × 3.65 mm^2^	≤40 × 40 mm^2^

**Table 2:** The most suitable ranges for the design parameters.

Design Parameters	Symbol	Suitable Range
Baffle Height	*h*	3.5 mm
Phototransistor-connected Resistance	*R*_1–4_	250–300 Ω
Emitter-mirror Distance	*z*	6.5–7.0 mm

**Table 3:** Performance of the proposed sensor.

	*x*-Axis	*y*-Axis
Range	±10°	±10°
Sampling rate	2000 Hz	2000 Hz
Average of errors	0.0265°	0.0265°
Standard deviation of errors	0.0251°	0.0159°
Resolution: range-to-error ratio	755	755
Mirror size	10 × 10 mm^2^	
PCB footprint	30 × 20 mm^2^	
